# Study on the Effects of Melatonin on Glycemic Control and Periodontal Parameters in Patients with Type II Diabetes Mellitus and Periodontal Disease

**DOI:** 10.3390/medicina57020140

**Published:** 2021-02-05

**Authors:** Diana-Maria Anton, Maria-Alexandra Martu, Marius Maris, George-Alexandru Maftei, Irina-Georgeta Sufaru, Diana Tatarciuc, Ionut Luchian, Nicoleta Ioanid, Silvia Martu

**Affiliations:** 1 Faculty of Dentistry, “Grigore T. Popa” University of Medicine and Pharmacy, 16 Universitatii Str., 700115 Iasi, Romania; diana_maria_a@yahoo.com (D.-M.A.); george.maftei.gm@gmail.com (G.-A.M.); irina_ursarescu@yahoo.com (I.-G.S.); ionut_luchian@yahoo.com (I.L.); nicole_ioanid@yahoo.com (N.I.); parodontologie1@yahoo.com (S.M.); 2 Faculty of Dental Medicine, “Titu Maiorescu” University, 67A Gheorghe Petrascu Str., 031593 Bucharest, Romania; 3 Faculty of Medicine, “Grigore T. Popa” University of Medicine and Pharmacy, 16 Universitatii Str., 700115 Iasi, Romania; diana.tatarciuc@gmail.com

**Keywords:** diabetes mellitus, periodontitis, melatonin, HbA1c

## Abstract

*Background and Objectives*: There is evidence that melatonin could improve the periodontal status and also glycemic control of patients with diabetes mellitus. Therefore, the aim of this study was to assess the effects of scaling and root planing plus adjunctive systemic treatment with melatonin on periodontal parameters and glycemic control in patients with type 2 diabetes and chronic periodontitis. *Materials and Methods*: The study was conducted on 54 subjects with periodontitis and diabetes mellitus randomly assigned to the study group (*n* = 27, subjects with scaling and root planing + melatonin) or control group (*n* = 27, subjects with scaling and root planing + placebo). Periodontal parameters (probing depth—PD; clinical attachment loss—CAL; bleeding on probing—BOP; and hygiene level) and glycated hemoglobin (HbA1c) were assessed at baseline and 8 weeks after. *Results*: At baseline, there were no significant differences between groups, but at the second evaluation 8 weeks later the association of melatonin with the non-surgical periodontal therapy exerted statistically significant improvements, both in periodontal parameters, with a significant decrease in periodontal disease severity, and glycated hemoglobin when compared to the control subjects. *Conclusions*: In our study, combined non-surgical periodontal treatment and systemic treatment with melatonin provided additional improvements to severe periodontal condition and the glycemic control of patients with diabetes type 2 when compared to non-surgical periodontal treatment alone.

## 1. Introduction

Periodontal disease is the result of chronic inflammatory manifestations, determined by the supra-gingival and sub-gingival accumulation of pathogenic biofilm. The inflammatory host response, with the loss of balance between bacterial aggression and the impaired ability of the immune system to cope with this aggression, leads to different forms of periodontal disease, from gingivitis to severe periodontitis [1]. The reported worldwide prevalence of periodontal disease varies from 45 to 50% in adults (superficial tissue impairment), to over 60% in elderly patients [2,3]. An important percentage of patients (around 11%) is affected by severe periodontal tissue breakdown [4], which can generate different forms of complications, ranging from loss of teeth to an affected quality of life [5]. Data from the literature also suggest that severe periodontal inflammation is linked to mortality [6].

Diabetes mellitus (DM) is a disease with an increased incidence worldwide; the number of patients under 14 years old diagnosed with insulin-dependent diabetes exceeds 500,000, while the number of adult diabetes patients is around 415 million. Moreover, it is estimated that there are about 193 million undiagnosed patients and 318 million people with an increased risk of developing DM during their lifetime [7]. It is estimated that, if this ascending trend is not stopped, by 2040 there will be 642 million people affected by diabetes [8].

The interrelation between periodontitis and diabetes was intensively studied; increased periodontal inflammation was associated with high serum levels of glycated hemoglobin (HbA1c) in subjects with diabetes and, interestingly, also in subjects without diabetes. It was observed that severe periodontal breakdown increases the risk of diabetes complications (vascular pathology, renal dysfunction) [9]. Furthermore, severe periodontal breakdown was associated with metabolic syndrome and increased oxidative stress in patients with type 2 DM [10]. Therefore, it was concluded that severe local inflammation (such as periodontitis) might exert an important role in the etiopathogenesis of diabetes and its complications [11], thus patients with diabetes and periodontal disease are more demanding regarding treatment options and efficacy.

The potential effect of non-surgical periodontal treatment (scaling and root planning—SRP) in periodontitis and DM subjects was considered. A systematic review observed a 0.36% reduction in HbA1c at 3 months after SRP [12].

Melatonin is a ubiquitous hormone which can also be found in the oral cavity. The salivary level of melatonin is about 1/4 to 1/3 of the serum level, varying from 1 to 5 pg/mL in daytime to 50 pg/mL during the night [13]. The presence of melatonin in saliva is considered to be the result of the passive passage in the salivary glands cells of serum unbound melatonin. Nevertheless, a study found the expression of arylalkylamine-N-acetyl-transferase (an enzyme involved in the night/day rhythmic production of melatonin) in the salivary glands of murine experimental models and human subjects [14]. The literature data confirm the presence of melatonin receptors in different oral sites, such as epithelial cells, osteoblasts, and fibroblasts [15], but the role of melatonin in the tissues of the oral cavity remains unclear.

Several studies have investigated the possible effects of melatonin on periodontitis subjects. A study observed the levels in gingival crevicular fluid and saliva in 70 patients with different periodontal pathologies. The researchers observed that the melatonin levels in severe periodontitis subjects were significantly lower than those in healthy or gingivitis subjects [16]. Regarding the influence of periodontal treatment, scaling and root planing exerted a positive effect by increasing the salivary melatonin levels in periodontitis subjects [17]. However, until now there has been no information regarding the improvement in the severe periodontal status of diabetic patients after non-surgical periodontal treatment and melatonin supplementation in the literature.

The drug supplementation of conventional etiologic periodontal treatment has been widely investigated, with a main focus on antibiotics and anti-inflammatory drugs. Nowadays, there is increased interest in less standardized drugs (such as sub-antimicrobial doses of doxycycline or omega-3 fatty acids), which could generate fewer adverse events and important local and systemic benefits. One of such therapies might also include melatonin intake. We hypothesized that melatonin supplementation, adjunctive to conventional non-surgical periodontal therapy, could improve the clinical periodontal status and decrease the HbA1c in patients with diabetes mellitus and periodontitis and prompt additional benefits to patients with severe periodontal disease. Therefore, the aim of this study was to assess the effects of scaling and root planing plus adjunctive systemic treatment with melatonin on periodontal parameters and glycemic control (HbA1c) in patients with type 2 diabetes and chronic periodontitis.

## 2. Materials and Methods

### 2.1. Inclusion and Design Criteria

In this double-blind, placebo-controlled, single-center study, 74 patients with type 2 diabetes with symptoms of periodontal disease were recruited. Subjects with fastening blood glucose levels higher than 126 mg/dL and glycated hemoglobin higher than 6.5% were defined as diabetic [18].

The study methodology was in accordance with ethical principles, including the Declaration of Helsinki from 2008; the study was conducted under the approval of the University of Medicine and Pharmacy “Grigore T. Popa” Iasi Ethics Committee nr. 30.07.2020. The subjects were informed regarding the study methodology and signed informed consent regarding their inclusion in the study.

The recruited subjects were thoroughly examined in order to assess the presence of periodontitis. Twenty patients out of 74 were excluded at baseline. Subjects who had undergone periodontal or anti-inflammatory treatment in the last 6 months, insulin treatment, significant change in drug use, and treatment of their diabetes or diet were excluded from the study. Other exclusion criteria were pregnancy, lactation, and smoking.

When performing a power analysis on the two analyzed groups with 90% power for a 1% difference in HbA1c between the two groups, a type I error of 0.05, and a type II error of 0.1, a total of 21 patients per group was needed. To anticipate the withdrawal rate of patients, an extra 25% was added to each group, thus the final patient number was 27 patients per group.

In conclusion, 54 subjects were randomly assigned to the study group (*n* = 27) or control group (*n* = 27) by a randomized block procedure.

### 2.2. Anthropometric Measurements

Anthropometric measurements, including height, weight, body mass index (BMI), waist circumference (WC), hip circumference (HC), and waist-to-hip ratio (WHR), were measured by a specialized nutritionist. The Nutritionist 4 software was used for the diet evaluation.

The intensity of physical activity was self-reported by questionnaire. We considered light activity to be 0–600 min/week (coefficient 3.3), moderate activity to be 600–3000 min/week (coefficient 4), and heavy activity to be >600 min/week (coefficient 8) [19].

### 2.3. Periodontal Status Assessment

One calibrated periodontal specialist performed the periodontal exam. The assessment of intra-examiner calibration was performed through test-retest exercises in ten subjects prior to the initiation of the study. For probing depth (PD), the intra-examiner predictability was 85%, and for clinical attachment loss (CAL) it was 82%. One more calibration exercise was performed during the study, with the predictability being 83% for PD and 81% for CAL.

The periodontal status was evaluated by measurements of bacterial plaque, bleeding on probing (BOP), periodontal probing depth (PD), and clinical attachment loss (CAL). For the BOP assessment, the gingival tissue was gently dried, the manual periodontal probe (Williams Novatech; Hu-Friedy, Chicago, IL, USA) was inserted in the depth of the gingival sulcus or periodontal pocket, and after 30 s the presence or absence of gingival bleeding was recorded. The oral hygiene was evaluated based on the presence of soft or mineralized deposits on the teeth. PD and CAL were measured at six sites per tooth (mesial-vestibular, central-vestibular, distal-vestibular, mesial-oral, central-oral, and distal-oral) with a manual periodontal probe (Williams Novatech; Hu-Friedy, Chicago, IL, USA). The probing depth was considered the distance between the gingival margin and the base of the gingival sulcus/periodontal pocket; CAL was considered the distance between the enamel-cement junction to the base of the sulcus/pocket. Severe periodontitis was acknowledged in subjects with CAL values ≥ 5 mm (not on the same tooth), moderate periodontitis in subjects with CAL values of 3–4 mm (not on the same tooth), and superficial periodontitis in subjects with CAL of 1–2 mm (not on the same tooth) [20]. The measurements were conducted at baseline (T0) and after 8 weeks (T1).

### 2.4. Treatment Methods

Patients were instructed to follow the nutritional recommendations and continue their usual physical activity throughout the study. All the patients in the study group and those in the control group received non-surgical periodontal debridement that involved ultrasonic scaling (Woodpecker UDS-A-LED, Guilin Woodpecker Medical Instrument Co., Ltd., Guilin, China) and manual root planing (Gracey Standard and Mini curettes—Hu-Friedy, Chicago, IL, USA) (SRP) in one session.

Instructions for dental hygiene were also provided, such as tooth brushing and flossing. Patients were instructed to avoid the use of mouthwash or other antiseptic oral products.

In addition to the SRP methods, subjects in the study group received two melatonin tablets (250 mg) containing 3 mg of melatonin, and the control group subjects received two placebo tablets (250 mg) for 8 weeks, 1 h before bedtime. The tablets were taken by direct ingestion with an appropriate quantity of water. There were no visual or tasting differences between the placebo tablets and the melatonin tablets. The potential adverse effects were closely monitored. The subjects who consumed less than 90% of the tablets, either from the study or the control group, were excluded from the study.

### 2.5. Glycated Haemoglobin Measurements

Glycated hemoglobin A1c (HbA1c) was determined for each patient. The method for determining HbA1c was immunoturbidimetric (Boehringer Mannheim, Mannheim, Germany), using a test with a high specificity of anti-HbA1c antibodies considered not to interfere with other forms of hemoglobin.

### 2.6. Statistical Analysis

The data are presented as mean ± standard deviation (SD). The Kolmogorov–Smirnov test was used to assess the data distribution. The independent *t* test was used for the evaluation of the statistical significance between groups at different time points.

## 3. Results

All the data from this study had a normal distribution. Fifty-four patients were initially recruited for the study. Four subjects were not able to complete the prescribed drug therapy and were excluded from the study. In total, 50 subjects (study group *n* = 25; control group = 25) completed the study.

The mean age of the subjects in the intervention and control group was 53.24 ± 3.4 and 52.21 ± 3.1 years, respectively. No significant differences (*p* ≥ 0.05) were observed in terms of demographic characteristics, physical parameters, duration of diabetes, food components, and drugs between the two groups at T0 (Table 1). No adverse effects of melatonin were observed during the study.

Melatonin therapy significantly decreased the mean values of PD and CAL in the study group after treatment completion (*p* < 0.001); singular SRP therapy also resulted in decreases in the control group but without reaching the level of statistical significance (Table 2). Both the bacterial plaque index and the gingival bleeding index showed significantly lower values for both study groups at T1, although the decreases were more significant for subjects receiving melatonin therapy.

Regarding the periodontitis severity, there were no significant differences between the study groups at baseline. Significant changes were observed for all severity categories (superficial, moderate, and severe) in the study group after 8 weeks, while in the control group we observed a slight decrease in the number of teeth with moderate and severe periodontitis (*p* > 0.05) and a significant increase in the number of teeth with superficial periodontitis (*p* < 0.05) (Table 2).

At T1, we noticed that conventional scaling and root planing therapy generated improvements in glycemic control, quantified by HbA1c measures, and the differences were significant for the intervention group, which also followed melatonin therapy (Table 2).

## 4. Discussion

Diabetes represents a major global concern due to its severe complications, such as vascular, renal, and neurologic pathology, as well as high risk of infections and impaired wound healing that increase the morbidity and mortality in DM patients [21]. Periodontitis was considered the sixth individual complication of DM due to complex patho-physiological inflammatory interactions [22]. The presence of periodontitis and, more importantly, of severe periodontal lesions such as alveolar bone destruction leading to tooth loss, in adjunction to a poor response to periodontal classical treatment, is frequent in diabetes subjects. Therefore, setting up an appropriate treatment plan becomes more than a necessity.

Several studies have shown the positive effects of melatonin and its physiological and pathological implications in the oral cavity [23,24]. A study on murine experimental periodontitis model reported that melatonin induced beneficial effects on inflammatory periodontal lesions [23]. It was demonstrated that melatonin decreases the number of osteoclasts in DM and periodontitis murine models, improving the alveolar bone loss and periodontal parameters, but it exerted no influence in systemic healthy periodontitis rats [25].

Our study demonstrated favorable changes for all the investigated periodontal parameters at 8 weeks from baseline in patients receiving scaling and root planing alone but statistical significance was achieved only for the plaque index and gingival bleeding. Our results showed that the administration of melatonin for 2 months significantly decreased the mean values of BOP, PD and CAL post-intervention. This is in line with the results of previous studies. Cutando observed that local delivery of melatonin significantly reduced gingival index and PD [26] while, in a previous study, it inhibited the pro-inflammatory markers in DM patients [27]. Similar findings were obtained by a study group led by Bazyar and Javid in an interventional study with systemic supplementation of melatonin [28]. Their research included also a potential explanation of the mechanisms involved in the beneficial effects of melatonin in diabetes subjects, with a decrease in inflammatory and oxidative stress markers [28,29].

Therefore, our study supports the possible beneficial effects of melatonin supplementation on the clinical markers of periodontal inflammation and periodontal tissue breakdown in diabetes mellitus patients. Moreover, we observed a significant reduction in the number of severe and moderate periodontitis teeth in subjects following melatonin treatment, in favor of an increased number of superficial periodontitis teeth. Even if the conventional non-surgical periodontal therapy alone followed the same trend, the difference was significant only for superficial periodontitis teeth. Consequently, we can assert that melatonin improves the severity of periodontitis in diabetes patients, bringing additional benefits for these patients.

Periodontal disease represents an infectious-inflammatory disease whose main determinant factor still remains bacterial periodontal pathogens, such as *Porphyromonas gingivalis*, *Treponema denticola*, or *Tannerella forsythia*. The interactions between the bacteria and the host can be modulated by a high diversity of local and systemic risk factors. The general literature data reports no important differences regarding the pathological biofilm between healthy and DM patients. A few heterogenic studies demonstrated a link between the glycemic control and shifts in periodontal biofilm [30]. Our study showed that both singular SRP and SRP + melatonin intake lowered the microbial plaque, but the decrease was more important when melatonin was added. A small number of studies focused on the potential antibacterial effect of melatonin, such as the one conducted by Srinath [31], but the subject still remains controversial.

The presence of DM does not necessarily represent an absolute indicator for periodontitis but the risk of periodontal disease may be higher in patients with diabetes who have poor glycemic control than in patients with well-controlled diabetes [32]. On the other side, a high number of studies demonstrated the favorable effect of non-surgical periodontal therapy [33]. In our study, scaling and root planing alone generated a decrease in terms of HbA1c, but the difference did not reach a statistic threshold, possibly to the short follow-up period.

A prospective study on 2973 systemic healthy subjects investigated the changes in HbA1c across a 5-year period [34]. The subjects with severe forms of periodontal tissue breakdown showed a HbA1c value approximately five-fold higher than the periodontal healthy subjects. This was the first study that proved the direct influence of periodontal inflammation on the HbA1c variations. In our study, we also observed a decrease in terms of HbA1c at 8 weeks after scaling and root planing, but the difference was statistically significant only for the subject group who also followed melatonin therapy.

Other studies validated the valuable influence of non-surgical periodontal therapy on improving glycemic control by measures of HbA1c [35,36,37]. The HbA1c reductions found after conventional periodontal therapy are mainly in the range of 0.27–0.48% [38], but there are no sufficient data regarding the maintenance in time of such values. Glycated hemoglobin represents a more reliable indicator for glycemic control than fastening glucose level; high values of glycated hemoglobin are correlated to severe DM complications, and even small reductions in its values mark down the morbidity and mortality from diabetes. It is considered that non-surgical periodontal treatment can exert such reduced HbA1c values as those obtained by adding a drug to the standard pharmacological regimen [39]. Although the recommended levels of HbA1c are <7%, the evidence indicates that there may not be a “safe” threshold for HbA1c. New diabetes treatment strategies are needed to address this growing public health problem.

The present study investigated the effects of standard non-surgical periodontal therapy plus adjunctive systemic treatment with melatonin on periodontal parameters and glycemic control (HbA1c) in patients with type 2 diabetes and chronic periodontitis over a period of 8 weeks. Further research is necessary to investigate the benefits of such therapy over a more prolonged period of time both in serum and saliva, as well as the possibility of higher systemic and local benefits of alternative methods of melatonin intake, such as sucking or chewing forms of melatonin tablets.

The results of our study are in line with the particular direction of the management of complex cases in an integrative manner. The paradigm of “periodontal medicine” is not new, but extensive research is currently performed in order to improve the inter-disciplinary medical approach to patients with systemic conditions and periodontal impairment. Melatonin might represent a highly potent drug in patients with periodontitis, and its beneficial effects could be even of greater importance in patients who present systemic pathologies, such as diabetes mellitus. Moreover, the melatonin regimen proved to be safe, without any adverse effects. We can conclude that a systemic therapy scheme with melatonin might be useful in improving the periodontal status of patients with diabetes and periodontitis by exerting favorable effects not only on local periodontal tissues but also on glycemic control, thus preventing severe complications of both pathologies.

## Figures and Tables

**Table 1:** Group characteristics at baseline.

Parameter		Control Group (*n* = 25)	Study Group (*n* = 25)	*p* Value
Age (years)		52.21 ± 3.1	53.24 ± 3.4	0.4
Gender (%)	Female	40%	44%	0.12
	Male	60%	56%	0.1
Height (cm)		166.20 ± 7.23	168.42 ± 6.99	0.2
WC (cm)		103.31 ± 6.21	102.45 ± 6.45	0.17
HC (cm)		108.22 ± 5.22	107.36 ± 6.08	0.09
WHR		0.95 ± 0.09	0.95 ± 0.07	0.9
Physical activity (minutes)		350.44 ± 120.20	322.62 ± 130.32	0.1

WC = waist circumference; HC = hip circumference; WHR = waist/hip ratio; values are expressed as mean ± standard deviation; *p* < 0.05 was considered statistically significant.

**Table 2:** Variation in periodontal parameters and glycated hemoglobin values.

	Control Group (*n* = 25)		Study Group (*n* = 25)		*p*0	*p*1
	T0	T1	T0	T1		
Periodontal parameters						
PD (mm) (Mean ± SD)	4.53 ± 1.01	4.40 ± 1.02	4.65 ± 1.04	2.27 ± 0.7	0.15	<0.001
*p* Value	0.12		<0.001			
CAL (mm) (Mean ± SD)	3.02 ± 0.93	2.98 ± 0.96	3.05 ± 0.56	1.24 ± 0.45	0.1	<0.001
*p* Value	0.08		<0.001			
Plaque index (+) (%)	100	48	100	24	0.9	0.07
*p* Value	<0.05		<0.001			
BOP (+) (%)	100	40	100	20	0.9	0.09
*p* Value	<0.05		<0.001			
Periodontitis severity *						
Superficial	168	191	174	257	0.24	<0.001
*p* Value	<0.05		<0.001			
Moderate	257	232	252	202	0.81	<0.05
*p* Value	0.08		<0.05			
Severe	72	74	76	53	0.64	<0.05
*p* Value	0.72		<0.05			
HbA1c (%)	7.6137 ± 0.62	7.5823 ± 0.57	7.6243 ± 0.71	6.2781 ± 0.31	0.738	<0.001
*p* Value	0.17		<0.001			

T0 = evaluation at baseline; T1 = evaluation after 8 weeks; PD = probing depth; CAL = clinical attachment loss; BOP = bleeding on probing index; HbA1c = Glycated hemoglobin; SD = standard deviation; *p*0 = *p* Value between groups at baseline; *p*1 = *p* Value between groups at 8 weeks; *p* < 0.05 was considered statistically significant. * Severe periodontitis: CAL values ≥ 5 mm, moderate periodontitis: CAL values of 3–4 mm, superficial periodontitis: CAL of 1–2 mm; values expressed as number of teeth.

## Data Availability

The data used to support the findings of this study are available from the corresponding author upon reasonable request.
